# Supplementary material for: Self-cleaning and surface chemical reactions during hafnium dioxide atomic layer deposition on indium arsenide
Source: Nat Commun. 2018 Apr 12;9:1412. doi: 10.1038/s41467-018-03855-z (PMC5897406; doi:10.1038/s41467-018-03855-z)
Supplement: Supplementary file 1 — Supplementary Information [file 41467_2018_3855_MOESM1_ESM.pdf]

Supplementary Information:

Self-cleaning and surface chemical reactions during HfO_2 atomic layer deposition on InAs

Timm et al.

content:

- Supplementary Figures 1 to 8
- Supplementary Table 1
- Supplementary Notes 1 to 6
- Supplementary References

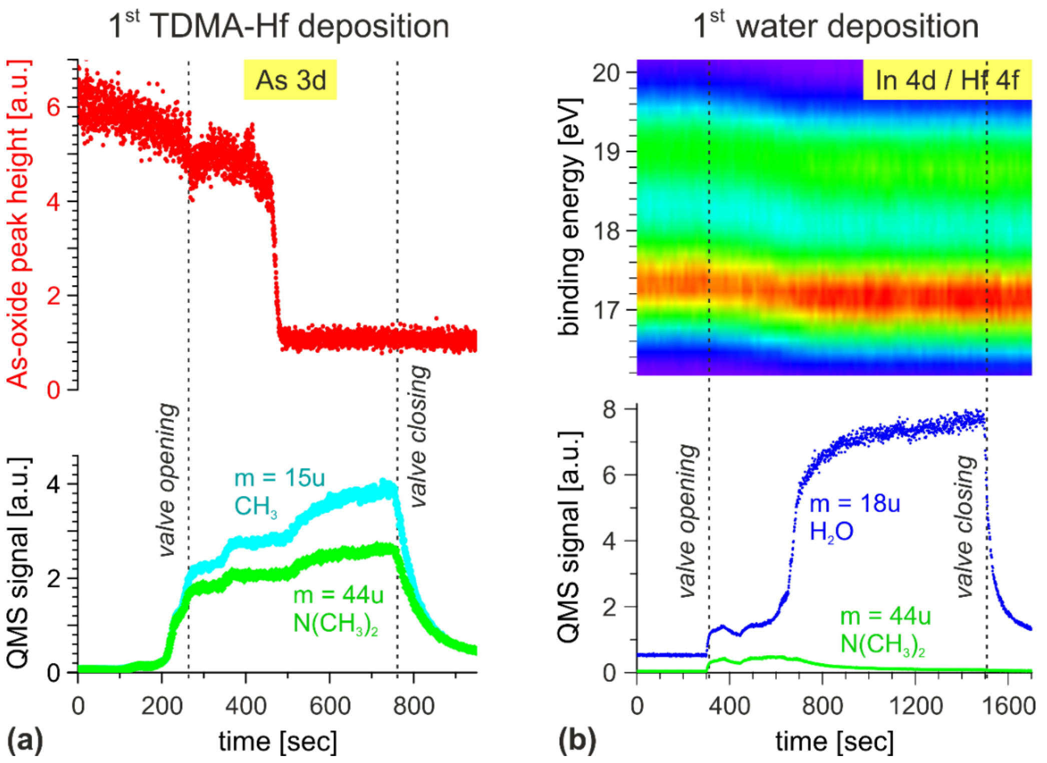

Supplementary Figure 1 | Reaction time and precursor pressure. XPS signals were acquired simultaneously with quadrupole mass spectrometer (QMS) data. (a) Time evolution of the XPS peak intensity at a binding energy of 44.6 eV, corresponding to the maximum of the As-oxide peak (top, red curve), and of the QMS signals corresponding to CH_3 (bottom, light blue curve) and $N(CH_3)_2$ (bottom, green curve) during exposure to TDMA-Hf. (b) Time evolution of overlapping Hf 4f and In 4d XPS core-level spectra (top), together with QMS signals corresponding to water (bottom, blue curve) and $N(CH_3)_2$ (bottom, green curve) during water exposure. XPS data has been obtained at photon energies of 320 eV (a) and 300 eV (b). Please refer to Supplementary Note 1 for further discussion.

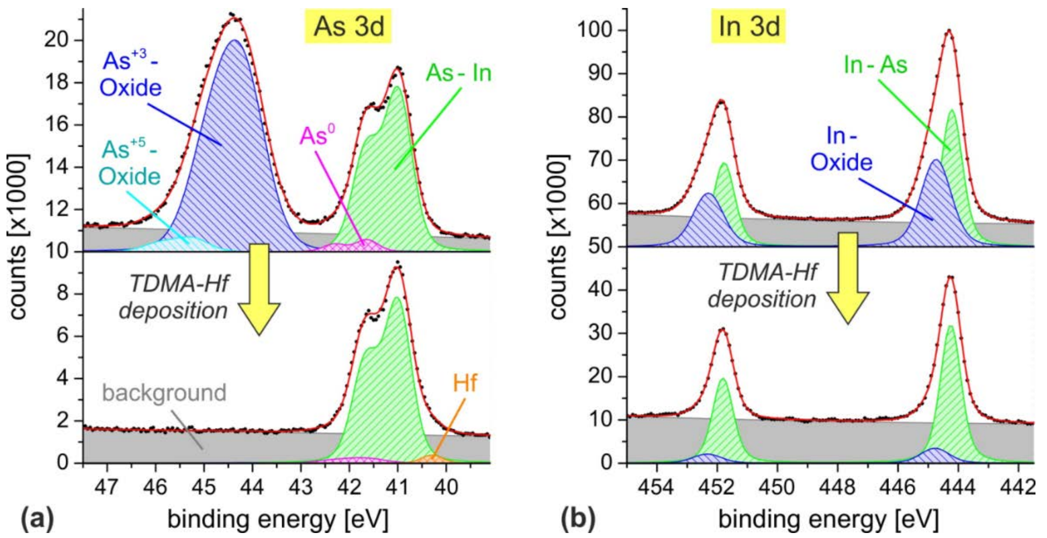

Supplementary Figure 2 | Self-cleaning effect of TDMA-Hf. XP spectra of the As 3d (a) and In 3d (b) core-levels, obtained under UHV conditions at photon energies of 170 eV (a) and 570 eV (b), before (top) and after (bottom) the first TDMA-Hf deposition at a sample temperature of 200°C. Experimental data (black dots) and fitted curves (red lines) are shown together with different fitted components, as indicated. A complete removal of the As-oxides and a strong reduction of the amount of In-oxides can be observed. In (a), bottom, a small contribution from the Hf 5p 1/2 core-level overlaps with the As 3d data. Count rates are normalized to show the same As-In (In-As) peak height before and after deposition.

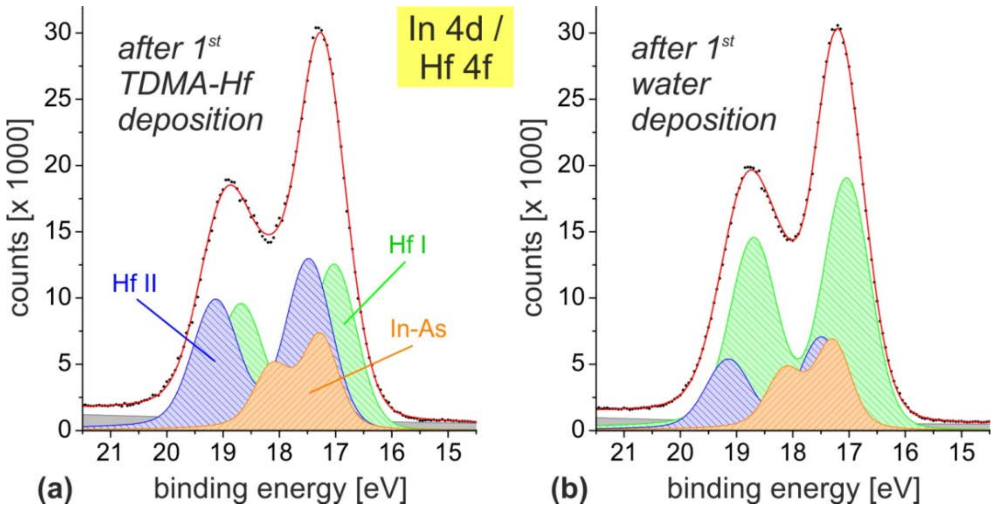

Supplementary Figure 3 | Hf 4f / In 4d XP core-level spectra. High-resolution spectra obtained under UHV conditions after TDMA-Hf deposition (a) and after water deposition (b), at a photon energy of 300 eV. Several fitted components are indicated.

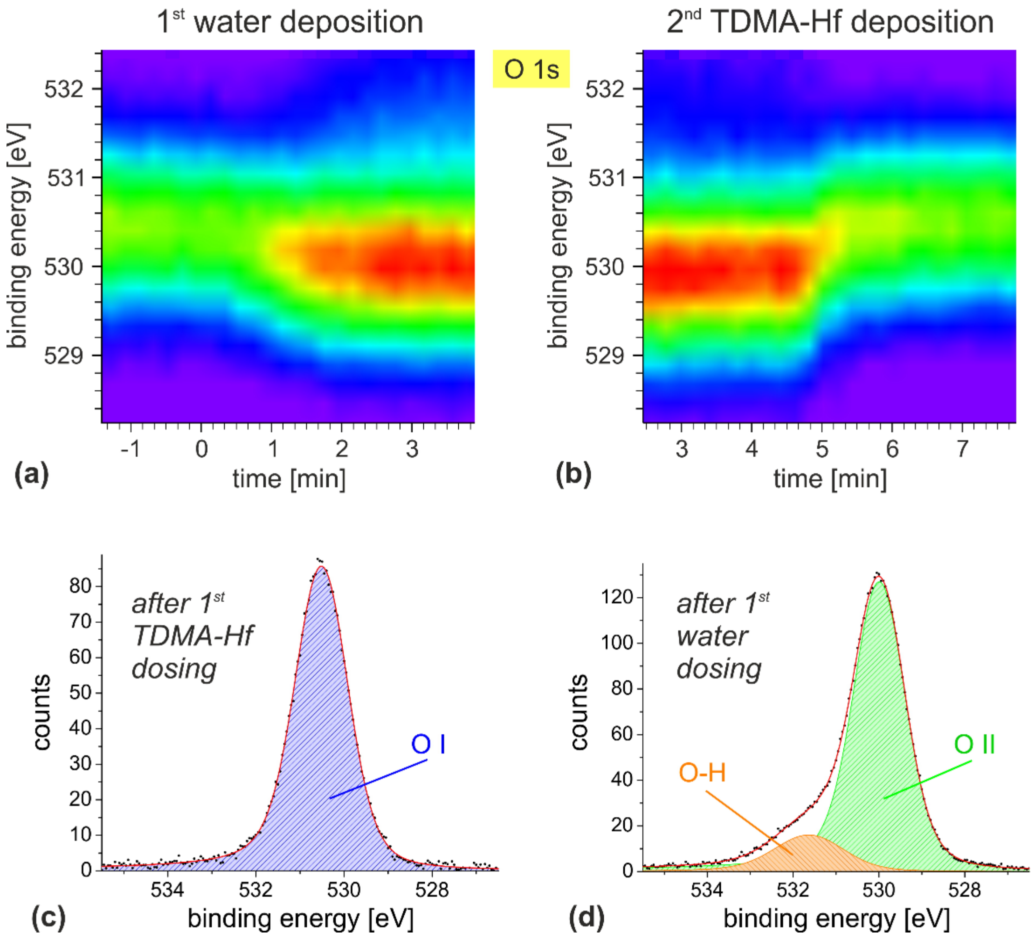

Supplementary Figure 4 | O 1s XP core-level spectra. (a,b) Time evolution of O 1s spectra during initial half-cycles of the ALD reaction, i.e. first water deposition (a) and first deposition of TDMA-Hf (b), at gas pressures of about 1 Pa and a sample temperature of 220°C. The time scales correspond to the opening of the gas inlet valve. (c,d) O 1s spectra obtained under UHV conditions after TDMA-Hf dosing (c) and after water dosing (d). Spectra have been obtained at a photon energy of 660 eV. Please refer to Supplementary Note 4 for further explanation.

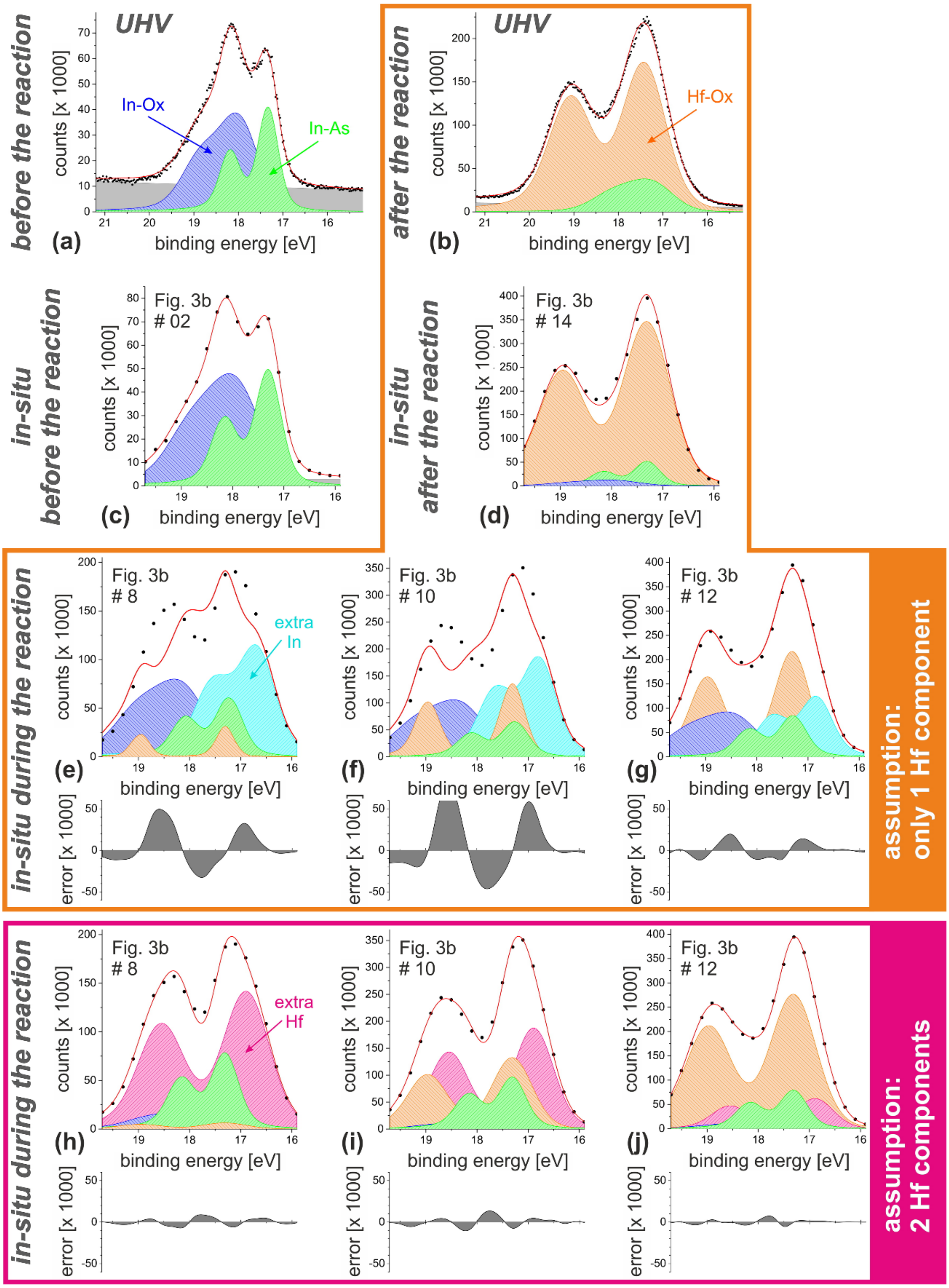

Supplementary Figure 5 | Hf 4f and In 4d contributions to XP core-level spectra. (a,b) High-resolution spectra obtained under UHV conditions (a) before and (b) after TDMA-Hf deposition. Fitted components are indicated. (c-j) AP-XP spectra obtained (c) at the beginning, (d) at the end, and (e-j) during the reaction upon TDMA-Hf deposition. These spectra are shown in Fig. 3b of the main manuscript, the sweep numbers are indicated. Spectra in (b,d-g) are fitted with only one Hf component, which does not shift in binding energy, while in (h-j) an

extra Hf component (pink) has been used for fitting. The bright blue doublet in (e-g) is an additional In component. Please refer to Supplementary Note 5 for further explanation. The difference between fitted results (red curves) and experimental data (black points) is shown as error (grey) in (e-j). Photon energies are (a,b) 300 eV and (c-j) 330 eV. The thickness of the Hf-containing layer deposited by ALD is expected to be less than 0.2 nm.

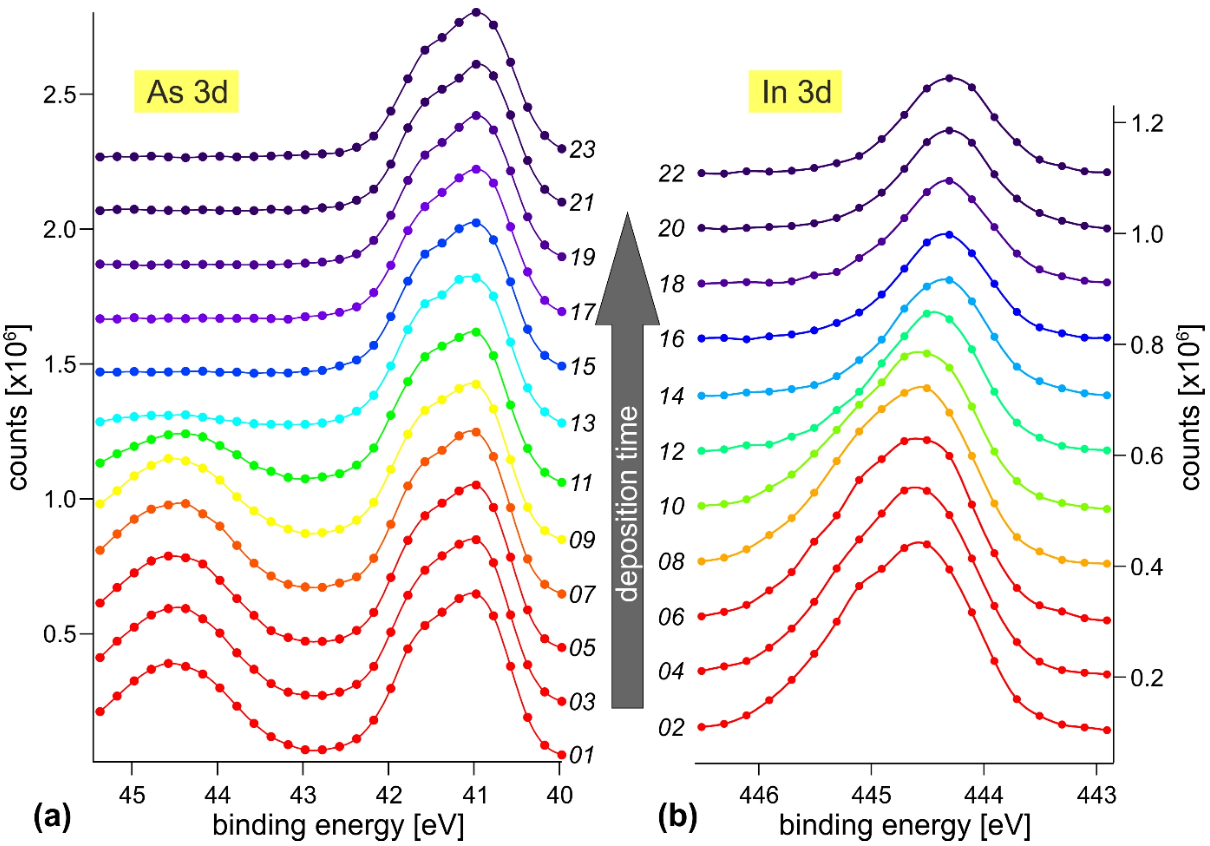

Supplementary Figure 6 | Correlation of In- and As-oxide removal. Correlated time evolution of (a) As 3d and (b) In 3d core level spectra, obtained alternately during TDMA-Hf deposition of the first ALD half-cycle, as indicated by the sweep index labels on each spectrum. As- and In-oxide removal can be correlated for spectra shortly before (red), during (orange, yellow, green, cyan, blue, purple), and after (dark purple) the reaction. Spectra have been obtained at a photon energy of 570 eV, a gas pressure of about 0.8 Pa, and a sample temperature of 220°C. The In 3d spectra are also shown as Fig. 3c in the main document.

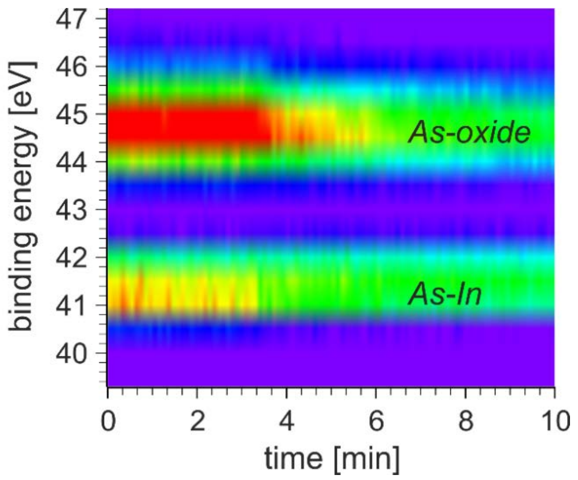

Supplementary Figure 7 | Incomplete ALD reaction at 180ºC. Time evolution of As 3d XP core level spectra during exposure to TDMA-Hf at a sample temperature of 180°C and a gas pressure of about 0.4 Pa, with a photon energy of 320 eV. Components corresponding to As-oxide and to As bound to In are indicated.

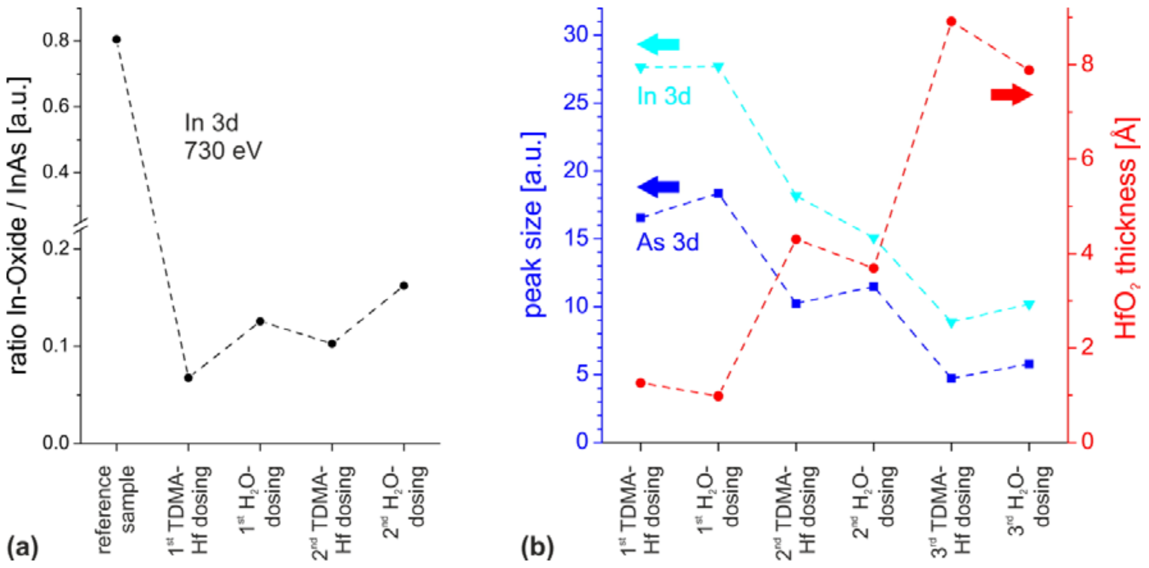

Supplementary Figure 8 | InAs signal and HfO_2 thickness upon subsequent ALD cycles. (a) Ratio between the size of the XPS peak corresponding to In-oxide and that corresponding to In-As, obtained by fitting corresponding components in experimental In 3d core-level spectra at a photon energy of 730 eV, for a reference sample and after subsequent ALD half-cycles. (b) Fitted peak sizes (left axis) of the InAs bulk component in As 3d spectra at a photon energy of 170 eV (blue curve) and of the InAs bulk component in In 3d spectra at a photon energy of 730 eV (light blue curve), and evaluated thickness of the deposited HfO_2 layer (red curve, right axis), after subsequent ALD half-cycles. All spectra were obtained in UHV conditions between the ALD half-cycles with a sample temperature of 220°C. Please refer to Supplementary Note 6 for further explanation.

<table>
<tr><th>condition</th><th>core-level</th><th>photon energy [eV]</th><th>mono-chromator exit slit [µm]</th><th>beamline broadening [eV]</th><th>pass energy [eV]</th><th>analyser entrance slit [mm x mm]</th><th>analyser broadening [eV]</th><th>total instrumental broadening [eV]</th></tr>
<tr><td rowspan="5">reaction cell</td><td rowspan="2">As 3d</td><td>320</td><td rowspan="5">100</td><td>0.12</td><td>20</td><td rowspan="5">3 x 20</td><td>0.35</td><td>0.37</td></tr>
<tr><td>570</td><td>0.23</td><td>20</td><td>0,35</td><td>0.42</td></tr>
<tr><td>In 3d</td><td>570</td><td>0.23</td><td>20</td><td>0.35</td><td>0.42</td></tr>
<tr><td>Hf 4f</td><td>300</td><td>0.11</td><td>50</td><td>0.88</td><td>0.89</td></tr>
<tr><td>O 1s</td><td>660</td><td>0.36</td><td>50</td><td>0.88</td><td>0.95</td></tr>
<tr><td rowspan="5">UHV</td><td>As 3d</td><td>170</td><td rowspan="5">50</td><td>0.03</td><td>20</td><td rowspan="5">1 x 20</td><td>0.22</td><td>0.22</td></tr>
<tr><td>In 3d</td><td>570</td><td>0.16</td><td>50</td><td>0.55</td><td>0.57</td></tr>
<tr><td>Hf 4f</td><td>300</td><td>0.06</td><td>50</td><td>0.55</td><td>0.55</td></tr>
<tr><td>O 1s</td><td>660</td><td>0.20</td><td>50</td><td>0.55</td><td>0.59</td></tr>
<tr><td>N 1s</td><td>530</td><td>0.15</td><td>20</td><td>0.22</td><td>0.27</td></tr>
</table>

Supplementary Table 1 | Experimental XPS parameters and instrumental broadening

Supplementary Note 1: Delay in observed reaction onset and long duration time of the chemical reaction

A several minutes long delay can be seen for all first ALD half-cycles, i.e. during all TDMA-Hf dosing, between the opening of the gas inlet valve and the start of the surface chemical reaction, as detected in the time-resolved AP-XP spectra. This delay amounts to about 2 minutes in Fig. 1 of the main manuscript, obtained at a TDMA-Hf pressure of about 1 Pa, and to about 13 minutes in Fig. 2a and Fig. 2c, both obtained at a pressure of about 0.3 Pa. Generally, lower gas pressures resulted in longer delay times.

The most direct explanation of this behaviour would be an actual delay of the chemical reaction like a kind of temporal activation barrier that needs to be overcome, due to the low pressure of the TDMA-Hf precursor applied here. Although this explanation cannot be excluded, it seems to be strongly unlikely, since a chemical mechanism resulting in such long delay times is hard to imagine.

In addition to this delay, also the duration of the chemical reaction is unexpectedly long: It amounts to several tens of seconds during the first ALD half-cycle (TDMA-Hf dosing), as shown in Fig. 1b and Fig. 2a,c of the main document as well as Supplementary Fig. 1a, and up to several minutes during the second ALD half-cycle (water dosing), which can be seen in Fig. 2b of the main document as well as in Supplementary Fig. 1b. Even though the pressure of the precursor gases used here is one to two orders of magnitude smaller than in commercial ALD setups under typical HfO_2 ALD conditions, this difference in pressure is not sufficient to explain the long duration.

Examples of slow ALD reactions can be found in literature: McDonnell et al. have reported on ALD of HfO_2 on MoS_2, where the TDMA-Hf precursor is not adsorbed on the MoS_2 surface but remains mobile after deposition and finally reacts with water molecules under the second ALD half-cycle without forming chemical bonds with the sample surface [1]. However, such a concept of mobile precursors or other examples of limited precursor adsorption during ALD [2], [3] might explain long time-scales, but not the combination of a long delay followed by a complete reaction afterwards, as seen here.

Quadrupole mass spectrometer (QMS) data obtained simultaneously with AP-XPS spectra allow some further insight in the experimental behaviour, as shown in Supplementary Fig. 1: The manual opening and closing of the gas inlet valve can clearly be seen in the QMS data. Since the gas flow was not completely constant and had to be re-adjusted by hand, some fluctuations of the gas pressure during the exposure of the sample occurred. The pressure increase in the reaction cell leads to a slight attenuation of the XPS signal, as can be seen in the measured peak height of Supplementary Fig. 1a. The onset and duration of the As-oxide reduction during exposure to TDMA-Hf however, as monitored by XPS, has no strong correlation with the QMS data. A different behaviour was observed during water exposure, as shown in Supplementary Fig. 1b: The chemical reaction, indicated by the shift in binding energy in the XPS spectra, starts immediately when the valve is opened. When the reaction is coming to an end after several minutes, i.e. when the position of the binding energy becomes constant, a strong increase in the QMS water signal can be seen, together with a further decline of the already weak $N(CH_3)_2$ signal.

We explain this behaviour by precursor adsorption in the tubes of the gas supply line and by the low vapour pressure of TDMA-Hf: The gas line of the AP-XPS systems with a total length of several meters was unheated and pumped to UHV conditions between subsequent ALD

half-cycles. Literature values for the vapour pressure of TDMA-Hf at room temperature vary between less than 1 Pa (datasheet by the supplier, Sigma-Aldrich Corporation) and 10 Pa [4]. When TDMA-Hf is supplied to the gas line, it can easily adsorb on the inner walls of the gas tubes, possibly with dissociation of some of the alkylamido or methyl groups. This can explain why both components appear with a similar intensity in the QMS data during the full time of the gas exposure. The large TDMA-Hf molecule is unfortunately too heavy to be detected by the QMS. When the gas tubes become saturated, an increasing, but initially still small amount of TDMA-Hf molecules can reach the surface, initiating the surface reaction which extends over about 20 s. After finishing TDMA-Hf deposition, the remaining gas is pumped out of the gas line, but an adsorbed layer on the tube walls will remain. When water is supplied to the gas line, the water signal of the QMS remains low for several minutes and increases strongly when the surface reaction comes to an end, although the total pressure in the reaction cell remains more or less constant. Thus, during the first phase of water exposure, the gas in the ambient pressure cell must have another main component than water. A possible explanation is that the water vapour entering the gas tube hits the Hf-containing adsorbate layer at the tube walls, undergoing a chemical reaction, where mainly the by-products of this reaction generate the pressure in the ambient pressure cell. In this case, the actual supply of water at the sample will be small, since the major supply of water reacts within the gas line, but not negligible, since water has a much larger vapour pressure than TDMA-Hf, thereby initiating a slow ALD reaction at the sample surface. When the adsorption or reaction of water on the walls of the gas tube saturates, the supply of water in the ambient pressure cell and thus at the sample surface increases, and soon the self-limiting ALD reaction is finished.

Supplementary Note 2: Oxide removal and self-cleaning effect evaluated from UHV-XP spectra

Before initiating the ALD process, and between each half-cycle deposition step, the sample surface was characterized by a set of XP spectra taken under UHV conditions, including As 3d, In 3d, In 4d / Hf 4f, O 1s, C 1s, and N 1s spectra at different photon energies.

Supplementary Figure 2 shows UHV XP core-level spectra obtained before and after the first TDMA-Hf deposition. The As 3d spectrum of the reference sample (Supplementary Fig. 2a) is dominated by a large surface oxide component, consisting of a major contribution from arsenic in a +3 oxidation state (like in As_2O_3) and a minor contribution from As+5 (like in As_2O_5 or $InAsO_4$), and also shows a small amount of arsenic in oxidation state 0 (corresponding to As-As bonds). After TDMA-Hf deposition, the As-oxide is removed below the XPS detection limit, and also the As^0 component has decreased in size, in good agreement with previous results of HfO_2 deposition on InAs [5], [6] and also with the self-cleaning effect observed on GaAs and InGaAs surfaces [7], [8], [9]. Water deposition and following Hf and water half-cycles do not significantly change the shape of the As 3d spectra. In the In 3d spectra, the large In-oxide peak of the reference sample is also strongly reduced in size after the first ALD half-cycle, as shown in Supplementary Fig. 2(b). Upon continuing ALD cycles, the measured XPS signal from the InAs gets more and more attenuated by the deposited HfO_2 film.

Supplementary Note 3: Hf 4f / In 4d XP core-level spectra

From time-resolved XP spectra of the overlapping Hf 4f and In 4d core-levels, as shown in Fig. 2 of the main document, a reversible shift upon subsequent ALD half-cycles was observed: The Hf 4f binding energies shift to lower values during water deposition and back to higher values during TDMA-Hf deposition. In order to investigate this shift, Supplementary Fig. 3 shows high-resolution XP spectra obtained under UHV conditions between the ALD depositions. The corresponding spectra have to be fitted by an In 3d doublet due to the underlying InAs substrate and by two Hf 4f doublets, separated in binding energy by 0.45 eV. The component with the Hf 4f 7/2 peak at 17.05 eV is dominant after water deposition, while the component at 17.5 eV increases in size upon TDMA-Hf deposition.

In principle, these two Hf 4f doublets correspond to two stable equilibrium configurations of the sample surface, which are reached between subsequent half-cycles. However, it should be noted here that it was unavoidable to have a small amount of water vapour present in the reaction cell, even upon exposure to TDMA-Hf. This small amount of water reacts with the sample surface, partially anticipating the reaction of the second ALD half-cycle. Therefore, the spectrum acquired after pumping down the cell to UHV conditions, plotted in Supplementary Fig. 3a, shows an equally large distribution of both Hf components, as the second half-cycle reaction had already started, though it is still far from being completed. Accordingly, the final state of the surface when finishing the TDMA-Hf deposition, as observed at the right edge of Fig. 2c in the main document, is not the same as the initial state of the surface when actively starting the water deposition, as seen at the left edge of Fig. 2b. On the contrary, TDMA-Hf precursor gas is only present in the reaction cell when the corresponding valve has been opened. The shift in binding energy observed in Fig. 2c, representing the full reaction of the first half-cycle, is therefore larger than the shift in Fig. 2b, where the initial part of the second half-cycle reaction is missing.

Supplementary Note 4: O 1s XP core-level spectra

AP-XP O 1s core-level spectra, monitoring the ALD half-cycle depositions, are shown in Supplementary Fig. 4a,b. Similar to the Hf 4f / In 4d spectra (shown as Fig. 2 in the main document), a reversible shift is observed, as the O 1s peak shifts to lower binding energies upon water deposition, together with an increase in peak intensity, and to higher binding energies upon TDMA-Hf deposition, together with a decrease of the peak intensity. In addition, a small shoulder at the high-energy side can be seen towards the end of the water deposition (Supplementary Fig. 4a). High-resolution XP spectra obtained under UHV conditions after TDMA-Hf dosing can well be fitted with only one component (Supplementary Fig. 4c), while spectra obtained in UHV after water dosing consist of a large component at a lower binding energy and an additional shoulder, which can be related to hydroxyl bonds (Supplementary Fig. 4d). The energy distance between both components amounts to 1.6 eV, in full agreement with literature values for O 1s oxide and hydroxide peaks in HfO_2 [10], [11].

Supplementary Note 5: Contribution of In 4d and Hf 4f components to the observed shifts in binding energy

A central finding of this work is the observed significant shift in binding energy of the Hf 4f spectra at the onset of Hf incorporation in the sample surface, as shown e.g. in Figs. 2d and 3b of the main document. As the Hf 4f signal overlaps with the In 4d core level, one might argue that the observed shift is not due to different chemical states of the Hf atoms, but is given by a chemical shift of the In 4d signal. Indeed, the removal of the native oxide on the InAs surface upon self-cleaning would decrease the intensity of the In-oxide component and shift the spectrum more towards the In-As component, which has a lower binding energy. Therefore, we have to investigate the influence of these In components on the combined In 4d/ Hf 4f spectra in detail, which we do here for the spectra obtained *in situ* during the TDMA-Hf deposition of the first ALD half-cycle, as shown in Fig. 3b of the main document. Thereby we have to take into account both the In 4d and the Hf 4f components, which overlap in binding energy. The thickness of the Hf-containing layer deposited by ALD is expected to be less than 0.2 nm, and the inelastic mean free path (IMFP) of the photoelectrons for the given conditions in the order of 0.7 – 0.8 nm. Since the XPS signal is dominated by contributions from the surface and decays exponentially for contributions from further inside the sample, such a short IMFP results in the Hf-containing top layer to contribute very strongly, while the influence of the InAs material underneath gets significantly attenuated already by a very thin top layer. In addition, the photoionization cross section of Hf 4f for the given photon energy is 7 to 8 times larger than that of In 4d (5.0 Mbarn for Hf 4f and 0,67 Mbarn for In 4d, at a photon energy of 300 eV, according to Supplementary Ref. [12]). Thus the signal from the Hf layer will be amplified by a factor of 7-8 compared to the In signal.

In the following, we *assume* that the observed shift is only due to changes in the In signal, and that the Hf signal always only consists of Hf-oxide bonds (as in HfO_2). We then *evaluate* if we can explain and fit the experimental results under this assumption. We start by investigating the UHV XP spectrum of the InAs surface with native oxide prior to the TDMA-Hf deposition, as shown in Supplementary Fig. 5a. Two In 4d doublets can well be fitted, with binding energies of the 5/2 peak of 17.3 eV and 17.93 eV and Gaussian full widths of half maximum (FWHM) of 0.49 eV and 1.03 eV, respectively. We assign these doublets to In-As and In-oxide. Considering the photon energy of 300 eV, which is not specifically surface sensitive, it is reasonable that both peaks appear with about the same height. Next, we fit the XP spectrum obtained under UHV conditions after the TDMA-Hf deposition, under the assumption that only one Hf doublet may be used. The result is shown in Supplementary Fig. 5b: A large Hf-oxide doublet with a 7/2 peak binding energy of 17.4 eV and a small In-As component are obtained, with no In-oxide component left. We used the same fitting parameters as stated in Table 1 of the main manuscript (Methods).

Now, we can turn to the AP-XP spectra obtained *in situ* during the TDMA-Hf deposition, as shown in Supplementary Fig. 5c-j. These spectra have a much lower energy resolution, and we therefore consistently use the peak binding energies obtained above and the same fitting parameters. At the beginning of the reaction, Supplementary Fig. 5c, the spectrum consists of the In-As and the In-oxide component and appears basically the same as under UHV conditions. It is important to note that in spite of the low energy resolution (see spacing of black points in Supplementary Fig. 5c), we obtain basically the same fit as in the case of the high-resolution UHV spectrum, showing the robustness of our fitting routines.

The area under both the In-As and the In-oxide curve amounts together to 139 units (which is an arbitrary value, which however can be used to compare relative changes during the reaction). After the reaction, Supplementary Fig. 5d, the spectrum can be fitted by the large Hf-oxide doublet, a small In-As component and a very small In-oxide component. The area under both In doublets amounts to 117 units. Supplementary Figure 5e-j shows XPS data obtained during the chemical reaction, where the strong shift in binding energy could be observed (green, cyan, and blue curves #08, #10, and #12 in Fig. 3b of the main manuscript). If we again assume that this shift is only due to the In signal, and that only one Hf component exists which remains constant in energy (i.e. that all Hf observed on the surface is Hf-oxide), a convergent fit can only be reached towards the end of the reaction – the best attempts are shown in Supplementary Fig. 5e-g: In order to fit the lower binding energy of the spectrum, we need to introduce an extra In doublet with a binding energy of the 5/2 peak of 16.7 eV, which is 0.6 eV below that of the In-As component observed before. This extra In component appears especially strong at the onset of the reaction (Supplementary Fig. 5e). In addition, we need to permit shifts in binding energy of the In-As and the In-oxide components in the range of ±0.3 eV. Adding even further In components does not improve the outcome of the fit. The area under the three In doublets amounts in total to 400 units in Supplementary Fig. 5e, 546 units in Supplementary Fig. 5f, and 447 units in Supplementary Fig. 5g, which in all three cases is much more than observed before the reaction. Even under these conditions, it is not possible to fit the shape of the spectrum reasonably well, especially in the range between 18 and 19 eV. This is further illustrated by the large error values (discrepancy between fitted results and experimental data) presented in Supplementary Fig. 5e,f. However, if we allow the existence of a second Hf component at lower binding energy – as discussed in the main manuscript – the spectra can well be fitted, as shown in Supplementary Fig. 5h-j. The binding energy of the second Hf doublet is 0.42 eV smaller than that of the Hf-oxide component. In these fits, the area under the two In components amounts to 130 units in Supplementary Fig. 5h, 141 units in Supplementary Fig. 5i, and 119 units in Supplementary Fig. 5j, which is very well in line with the values obtained before and after the reaction.

These results clearly show that the *assumption* of the observed shift in binding energy being due only to changes of the In components *does not hold* for three reasons: (1) A chemical shift from In-oxide to In-As is not sufficient to explain the observed binding energies, instead an additional In component is needed for curve fitting. Its binding energy of 0.6 eV below that of In-As cannot be motivated in the given material system. Various surface components have been reported in literature for clean, reconstructed InAs, but none of them has such a strong shift in binding energy [13]. In addition, we do not expect any surface reconstruction to form during the self-cleaning process. (2) Upon the onset of the reaction, a strong increase in the total intensity of the AP-XP spectrum is observed, which before has been attributed to the larger photoionization cross-section of Hf. However, in order to fit the energy shift of the obtained spectra by additional In components, the In signal has to increase significantly, reaching a value which is four times as large as before the reaction. Such a strong increase cannot be explained, since no additional In material is provided. Prior to the reaction, which includes the self-cleaning effect, the In-As signal is attenuated by the native oxide. This native oxide consists of both As-oxides and In-oxides and therewith also contributes to the In 4d signal through the In-oxide component. Therefore we compare the total In signal, including In-oxide, In-As, and the additional In component mentioned above. (3) The line shape of the AP-XP spectra obtained during the reaction clearly shows the signature of a doublet with a large spin-orbit splitting as that of Hf 4f. It cannot be fitted by dominant contributions from In 4d (as

it is the case in Supplementary Fig. 5e,f). In the contrary, the obtained spectra can very well be explained by the occurrence of an extra Hf component with a lower binding energy.

In our analysis we have applied peak fitting both by the standard Marquardt's algorithm [14] (using Igor) and by an algorithm for constrained minimization (using FitXPS), namely the quadratic tensor model (QTM) algorithm of Hanson and Krogh [15]. The QTM algorithm allows for one-sided or two-sided bounds on parameter values and for one-sided or two-sided bounds on linear constraints between parameters. Hanson and Krogh have shown that the QTM algorithm is particularly successful in solving badly conditioned problems (such as overlapping peaks and few measurement points). For fitting AP-XP spectra with few measurement points, the use of boundary conditions and constraints allows us the search parameter space while taking into account strong constraints such as the spin-orbit splitting and the branching ratio of a doublet peak or the Lorentzian FWHM. Even using a robust algorithm as QTM, that will search for solutions within boundaries and avoid many false minima [16], can still end in a false minimum if initial parameters are very far off. For this reason we also manually varied initial parameters such as the number of In doublets considered, there binding energy, and their intensity within physically reasonably values. Even using QTM and varying all parameters within conceivable limits, our experimental results cannot be fitted satisfactorily if we assume that the observed shift in binding energy is only due changes in the In signal.

Supplementary Note 6: Attenuation of the InAs signal and increasing HfO_2 thickness upon subsequent ALD half-cycles

In this section we present a more quantitative analysis of the UHV-XP spectra, starting with the As 3d and In 3d spectra obtained at the reference substrates, prior to any material deposition. By simulating the relative oxide and bulk peak sizes for each spectrum as described previously [5], [17], the thickness of the native oxide film is obtained, amounting to between 0.7 nm and 1.1 nm for different samples. Since the In substrates have been etched by HCl and subsequently transported through air prior to loading into the UHV chamber of the XPS setup, a relatively thin native oxide layer with small changes between different samples was expected.

The fact that all As-oxides are removed below the detection limit of the XPS setup (which is far below 0.1 monolayer), but a small amount of In-oxides remained after the initial TDMA-Hf dosing, shows that the interface between the InAs substrate and the HfO_2 layer consists of In-O-Hf bonds. This is in agreement with earlier results [5], and also with DFT studies for the GaAs material system, where Ga-O-Hf bonds were predicted [18]. By fitting the In 3d spectra taken after subsequent dosing of the ALD process, we find that the amount of interfacial In-oxide increases during the first two ALD full-cycles, as shown in Supplementary Fig. 8a. This demonstrates that the first cycle has not yet produced a full monolayer of HfO_2.

The area under the fitted curves of the InAs bulk peaks in both the As 3d and the In 3d spectra is decreasing upon subsequent ALD cycles, as shown in Supplementary Fig. 8b. This is due to an increasing attenuation of the InAs photoelectron signal with increasing thickness of the deposited layer on top. Interestingly, in most cases a slight increase of the peak area – i.e. a decrease of the attenuation – can be observed upon each second half-cycle, the dosing of water. The same trend is found for the peak sizes of the In-oxide peaks (Supplementary Fig. 8a), and it has been observed independently of the kinetic energy of the photoelectrons.

This finding might be due to an actual decrease of the deposited top-layer thickness upon water dosing, but since this dosing implies a chemical reaction at the surface, the observed behaviour could also be explained by a change of the chemical composition of the top-layer. In the latter case, a layer terminated by TDMA-Hf dosing would have a smaller electron inelastic mean free path (leading to stronger attenuation) than a layer terminated by water-dosing.

From the attenuation of the InAs signal upon ALD cycles it is possible to calculate the deposited layer thickness, according to

$$I = I_0 \exp\left(-d/\lambda\right),$$

where I is the photoelectron flux, I_0 is the flux before attenuation, λ is the electron inelastic mean free path, and d is the thickness of the deposited layer. Since neither the exact chemical composition of the top-layer nor its electron inelastic mean free path is known, we assume the top-layer to consist of HfO_2, as best possible approximation. As 3d and In 3d core-level spectra at photon energies of 170 eV, 320 eV, and 920 eV for As 3d as well as 570 eV and 730 eV for In 3d are used, corresponding to electron kinetic energies of 130 eV, 280 eV, and 880 eV. Electron inelastic mean free paths are calculated for these energies using the TPP formula [19], resulting in values of 5.1 Å, 7.2 Å, and 15.5 Å. Since the ALD deposition does not start with a clean InAs surface, but with a reference sample covered with native oxide, we first take into account the results of the native oxide thickness as discussed above and calculate a compensation factor for the attenuation of the InAs photoemission signal by the native oxide layer in the spectra of the reference sample. Changes in the photon flux between different spectra due to a slowly decaying electron beam in the synchrotron are corrected using tabulated values of the beam current. However, since the sample position, the monochromator settings, and the lens settings of the electron analyser are different for UHV XPS characterization and the *in situ* XPS measurements during the ALD deposition, these settings are changed back and forth for each ALD half-cycle, possibly resulting in different XPS intensities. In order to compensate these changes, we can use depth profiles, investigating the same core-level at different photon energies and thus different inelastic mean free paths, to calibrate the measured count rates. Results for the correspondingly obtained HfO_2 layer thickness upon subsequent ALD half-cycles are shown in Supplementary Fig. 8b.

Supplementary References

[1] McDonnell, S., Brennan, B., Azcatl, A., Lu, N., Dong, H., Buie, C., Kim, J., Hinkle, C. L., Kim, M. J. & Wallace, R. M., HfO2 on MoS2 by Atomic Layer Deposition: Adsorption Mechanisms and Thickness Scalability. *ACS Nano* **7**, 10354 (2013).

[2] Liu, H., Xu, K., Zhang, X. & Ye, P. D., The integration of high-k dielectric on two-dimensional crystals by atomic layer deposition. *Appl. Phys. Lett.* **100**, 152115 (2012).

[3] McDonnell, S., Pirkle, A., Kim, J., Colombo, L. & Wallace, R. M., Trimethyl-aluminum and ozone interactions with graphite in atomic layer deposition of Al2O3. *J. Appl. Phys.* **112**, 104110 (2012).

[4] Bartholomew, L., Barelli, C., Owyang, J., DiCarlo, R., Shenai, D., Marsman, C. & Senzaki, Y., Comparison of ALD of HfO2, SiO2, and HfxSi1-xO2 Thin Films Using Various Metal/Silicon Alkylamide Precursors and O3. *ECS Transactions* **3** (15), 37 (2007).

[5] Timm, R., Fian, A., Hjort, M., Thelander, C., Lind, E., Andersen, J. N., Wernersson, L.-E. & Mikkelsen, A., Reduction of native oxides on InAs by atomic layer deposited Al2O3 and HfO2. *Appl. Phys. Lett.* **97**, 132904 (2010).

[6] Timm, R., Hjort, M., Fian, A., Thelander, C., Lind, E., Andersen, J. N., Wernersson, L.-E. & Mikkelsen, A., Interface composition of atomic layer deposited HfO2 and Al2O3 thin films on InAs studied by X-ray photoemission spectroscopy. *Microelectron. Eng.* **88**, 1091 (2011).

[7] Hinkle, C. L., Sonnet, A. M., Vogel, E. M., McDonnell, S., Hughes, G. J., Milojevic, M., Lee, B., Aguirre-Tostado, F. S., Choi, K. J., Kim, H. C., Kim, J. & Wallace, R. M., GaAs interfacial self-cleaning by atomic layer deposition. *Appl. Phys. Lett.* **92**, 071901 (2008).

[8] Milojevic, M., Aguirre-Tostado, F. S., Hinkle, C. L., Kim, H. C., Vogel, E. M., Kim, J. & Wallace, R. M., Half-cycle atomic layer deposition reaction studies of Al2O3 on In0.2Ga0.8As (100) surfaces. *Appl. Phys. Lett.* **93**, 202902 (2008).

[9] Chang, Y. C., Huang, M. L., Lee, K. Y., Lee, Y. J., Lin, T. D., Hong, M., Kwo, J., Lay, T. S., Liao, C. C. & Cheng, K. Y., Atomic-layer-deposited HfO2 on In0.53Ga0.47As: Passivation and energy-band parameters. *Appl. Phys. Lett.* **92**, 072901 (2008).

[10] Hofmann, S. & Sanz, J. M., Quantitative XPS analysis of the surface layer of anodic oxides obtained during depth profiling by sputtering with 3keV Ar+ ions. *J. Trace Microsc. Tech.* **1** (3), 213 (1982-83).

[11] Lehan, J. P., Mao, Y., Bovard, B. G. & Macleod, H. A., Optical and microstructural properties of hafnium dioxide thin films. *Th. Sol. Films* **203**, 227 (1991).

[12] Yeh, J. J. & Lindau, I., Atomic subshell photoionization cross-sections and asymmetry parameters: 1<=Z<=103. *Atomic Data and Nuclear Data Tables* **32**, 1 (1985).

[13] Laukkanen, P., Punkkinen, M. P. J., Ahola-Tuomi, M., Lång, J., Schulte, K. *et al.*, Core-level shifts of the c(8x2)-reconstructed InAs(100) and InSb(100) surfaces. *J. Electron Spectrosc. Relat. Phenom.* **177**, 52 (2010).

[14] Marquardt, D. W., An Algorithm for Least-Squares Estimation of Nonlinear Parameters. *J. Soc. Ind. Appl. Math.* **11**, 431 (1963).

[15] Hanson, R. J. & Krogh, F. T., A quadratic-tensor model algorithm for nonlinear least-squares problems with linear constraints. *ACM Trans. Math. Softw.* **18**, 115 (1992).

[16] Adams, D. L., A simple and effective procedure for the refinement of surface structure in LEED. *Surface Science* **519**, 157 (2002).

[17] Timm, R., Hjort, M., Fian, A., Borg, B. M., Thelander, C., Andersen, J. N., Wernersson, L.-E. & Mikkelsen, A., Interface composition of InAs nanowires with Al2O3 and HfO2 thin films. *Appl. Phys. Lett.* **99**, 222907 (2011).

[18] Wang, W., Xiong, K., Wallace, R. M. & Cho, K., Impact of Interfacial Oxygen Content on Bonding, Stability, Band Offsets, and Interface States of GaAs:HfO2 Interfaces. *J. Phys. Chem. C* **114**, 22610 (2010).

[19] Tanuma, S., Powell, C. J. & Penn, D. R., Calculations of Electron Inelastic Mean Free Paths. *Surf. Interf. Anal.* **21**, 165 (1993).
